# Single Diameter Modulation Effects on Ni Nanowire Array Magnetization Reversal

**DOI:** 10.3390/nano11123403

**Published:** 2021-12-16

**Authors:** Luis C. C. Arzuza, Victor Vega, Victor M. Prida, Karoline O. Moura, Kleber R. Pirota, Fanny Béron

**Affiliations:** 1 Physics Institute “Gleb Wataghin”, Universidade Estadual de Campinas (UNICAMP), Campinas 13083-859, Brazil; luisarzuza179@gmail.com (L.C.C.A.); krpirota@ifi.unicamp.br (K.R.P.); 2 Physics Department, Universidade Federal da Paraíba (UFPB), João Pessoa 58051-900, Brazil; karoline.oliveira@academico.ufpb.br; 3 Physics Department, University of Oviedo, 33007 Oviedo, Spain; vegavictor@uniovi.es (V.V.); vmpp@uniovi.es (V.M.P.)

**Keywords:** magnetic nanowire, diameter modulation, magnetization reversal, interaction field, first-order reversal curves (FORC)

## Abstract

Geometrically modulated magnetic nanowires are a simple yet efficient strategy to modify the magnetic domain wall propagation since a simple diameter modulation can achieve its pinning during the nanowire magnetization reversal. However, in dense systems of parallel nanowires, the stray fields arising at the diameter interface can interfere with the domain wall propagation in the neighboring nanowires. Therefore, the magnetic behavior of diameter-modulated nanowire arrays can be quite complex and depending on both short and long-range interaction fields, as well as the nanowire geometric dimensions. We applied the first-order reversal curve (FORC) method to bi-segmented Ni nanowire arrays varying the wide segment (45–65 nm diameter, 2.5–10.0 μm length). The FORC results indicate a magnetic behavior modification depending on its length/diameter aspect ratio. The distributions either exhibit a strong extension along the coercivity axis or a main distribution finishing by a fork feature, whereas the extension greatly reduces in amplitude. With the help of micromagnetic simulations, we propose that a low aspect ratio stabilizes pinned domain walls at the diameter modulation during the magnetization reversal. In this case, long-range axial interaction fields nucleate a domain wall at the nanowire extremities, while short-range ones could induce a nucleation at the diameter interface. However, regardless of the wide segment aspect ratio, the magnetization reversal is governed by the local radial stray fields of the modulation near null magnetization. Our findings demonstrate the capacity of distinguishing between complex magnetic behaviors involving convoluted interaction fields.

## 1. Introduction

Several possible spintronics devices are based on the controlled propagation and manipulation of magnetic domain walls (DWs) along a ferromagnetic nanowire [1]. Among these, the domain wall race-track memory [2,3], where the binary information is recorded as the presence or absence of a magnetic DW, yielded extensive research on the DW behavior [4,5]. Specifically, due to their isotropic section, cylindrical magnetic nanowires have been proved to reach faster DW propagation velocities compared with magnetic stripes, without the Walker breakdown phenomenon [6]. Therefore, they have been used as simple systems to investigate different aspects of magnetic DW control through a heterogeneous medium. Since DW structure arises from an interplay between the exchange and magnetostatic energies, a simple yet efficient way to locally control the DW propagation is through the insertion of geometric discontinuities [7,8,9,10].

One way to experimentally achieve cylindrical magnetic nanowires is via nanoporous alumina templates obtained by two-step anodization [11], which constitute an ideal system to produce ordered nanomaterials. The easy control of the nanoporous geometry (length and diameter) makes them outstanding patterned templates for the fabrication of different types of nanomaterials [12,13]. Magnetic nanowire arrays embedded in nanoporous alumina membranes via a template-assisted method [14] are suitable for high-density (3D) magnetic recording, for example. For these cylindrical nanowires, the simplest geometrical modification consists in a diameter modulation, occurring more or less abruptly. Previous literature mainly focused on individual nanowires, both from experimental and simulation points of view [15,16,17,18,19]. The main findings are that a non-negligible stray field is created at the diameter modulation [20,21], with its geometry depending on the modulation shape [22,23], and that a DW pinning can occur at the modulation [24,25,26,27]. However, only a few studies were conducted up to now on diameter-modulated nanowire arrays, because of the encountered difficulties during their fabrication procedure and result analysis [20].

In this context, the first-order reversal curve (FORC) technique has already proved to be adequate to probe and analyze complex magnetic behavior [28], especially in ferromagnetic nanowire arrays [29,30,31,32,33,34,35,36,37], inclusively with diameter modulation [38]. We previously applied the technique to two Ni nanowire array geometries with narrow and wide diameters of 35 and 52 nm, respectively [39]. The results showed a major modification of the array magnetization reversal compared to a single-diameter reference array: instead of being governed by a unique mean demagnetizing interaction field, the array magnetic behavior seems to depend upon two competing mean interaction fields. They were identified as spatially localized at each nanowire extremity, whereas their strength was related to the extremity diameter. However, one of the investigated nanowire arrays exhibited an additional and unexpected FORC feature. This result suggested that, for some sets of nanowire geometric dimensions, the array magnetization reversal may be more complex than solely arising from two mean interaction fields. 

Here, the work aims to improve the understanding of the different magnetization reversal processes for an array of ferromagnetic nanowires with modulated geometry. Therefore, we performed a systematic study of Ni nanowire arrays presenting a single diameter modification. As a first step, the number of diameter modulation was limited to a unique one along the nanowires, to introduce in the nanowire system a single additional source of stray field (located at the diameter modification). Our goal is to investigate if the presence of neighboring nanowires influences the DW pinning and vice-versa, i.e., if neighboring nanowires with a pinned DW can modify the magnetization reversal of a nanowire. This was performed by exploring the influence of the length/diameter segment aspect ratio on the complex interaction field present in the array and its consequences on the nanowire magnetization reversal. 

The diameter modulation was made possible by a template-assisted synthesis approach based on nanoporous alumina membranes [14,40,41], fabricated by a combination of mild-anodization and pore widening steps [22,42], yielding the recently elaborated three-step anodization method [39]. It is a low-cost method that ensures high reproducibility and uniformity on large alumina template areas. Additionally, it produces a sharp transition between the narrow and wide segments [39]. The narrow segment was kept constant (35 nm diameter, 3.5 μm long), while either the diameter or the length of the wider segment was gradually increased. The analysis of their respective first-order reversal curves allows dividing the investigated systems into two main groups, depending on their magnetization reversal processes. The results suggest that, whereas the magnetization reversal effectively occurs through two different interaction fields when the nanowire array is nearly saturated, the diameter modulation stray field of the neighboring nanowires yields a more complex magnetic behavior. 

## 2. Materials and Methods 

We produced the nanowire arrays using the three-step anodization method. Together with an intermediate chemical etching step, it enables the fabrication of diameter-modulated nanopores in an alumina template. The obtained diameter-modulated nanowires are electrochemically grown embedded in a highly ordered hexagonal nanoporous alumina membrane, which serves as a template during the nanowire electrodeposition. The three-step anodization method for the patterned alumina template occurred under mild anodization conditions [11], which consists of an applied voltage of 40 V in 0.3 M oxalic acid electrolyte kept at 3 °C. The widening of the 35 nm diameter pores obtained during the second anodization step was done through an H_3_PO_4_ chemical etching. After the pore widening process, the samples were submitted to a third anodization step under the same electrochemical conditions as the second one, to grow the narrow nanopore segment. Both the anodization and pore widening times were calculated in order to obtain the desired nanowire geometry [39]. While the narrow segment remains identical for all nanopore arrays (diameter *d* = (36.0 ± 0.3) nm, around 105 nm interpore distance and length *l* = (3.4 ± 0.2) μm), either the wider segment length *L* varies between 2.5 and 10 μm (with a (52.0 ± 0.2) nm diameter), or its diameter *D* varies between 45 and 65 nm (with a (2.5 ± 0.3) μm length). Scanning electron microscopy (SEM) top and bottom micrographs of similar alumina templates obtained through the three-step anodization technique can be seen in Ref. [39].

Before the magnetic nanowire electrodeposition, we completely removed the alumina barrier layer, which is located at the nanopore bottom of the alumina template. This step is performed by removing the remaining Al substrate with a 14.4 mL CuCl_2_ + 200 mL HCl + 400 mL H_2_O solution, followed by a 5 wt.% H_3_PO_4_ solution at room temperature for 2 h. It both etches the bottom alumina barrier layer and yields opened pores on both extremities. We sputtered a thin layer of gold of approximately 16 nm (SC7620 Polaron, Hertfordshire, England) on the bottom alumina template surface (which presents the narrow pores) to close the openings. Gold electrodeposition from a commercial gold plating solution (Orosene 999, Technic, LO, Italy, applied voltage of 2.6 V) was performed afterward. It ensures a good and uniform electrical contact that will serve as an electrode for the charge transfers involved during the later nanowire electrodeposition. Subsequently, we filled the pores of these alumina templates via Ni potentiostatic electrodeposition at 35 °C with a voltage of −1.4 V vs. Ag/AgCl electrode with a commercial N5770A power supply (Keithley, Cleveland, OH, USA). The solution was a Watts-type electrolyte composed of NiSO_4_ (300 g/L), NiCl_2_ (45 g/L), and H_3_BO_3_ (45 g/L). These fabrication parameters result in a high filling factor and a small nanowire length distribution. Scanning electron microscopy (Inspect F-50 High Resolution, FEI, Hillsboro, OR, USA) was employed to characterize the morphology of the magnetic modulated nanowires embedded in porous alumina membranes.

The magnetic characterization was performed at room temperature, with a magnetic field applied along the nanowire axis. For each array, a set of 100 to 120 first-order reversal curves (FORCs) was acquired on a vibrating sample magnetometer (Lakeshore, Westerville, OH, USA), according to the following sequence: after fully saturating the array by applying 10 kOe, the field is swept down until a reversal point (*H_r_*), before measuring the magnetization *M* while increasing back the applied field (*H*) until the saturation. To ensure an adequate resolution of the FORC results while optimizing the acquisition time, the magnetic field intervals ΔH and ΔH_r_ were maintained at 25 and 50 Oe, respectively [30], yielding a total of around 10,000 to 15,000 data points per measurement. The FORC distribution is calculated through a mixed derivative of the magnetization in function of both *H* and *H_r_* [43]. We used a Shepard method for bivariate interpolation due to its robustness against artifacts arising from irregular data grids [44]. For each data point, in order to not artificially flatten and distort the FORC distribution, the interpolation was performed taking into account the minimum possible value of first neighbors yielding an acceptable noise level [31,32]. We used a value of 100 for all arrays, except those with *L* ≥ 5.6 μm for which it was possible to decrease down to 60 first neighbors due to their higher magnetic signal. Furthermore, to minimize the artifacts created by the absence of the *H* < *H_r_* data points, which may hide some FORC features located near the *H* = *H_r_* region, the FORC curve beginnings were extrapolated minimizing the discontinuities near *H* = *H_r_* [45].

The resulting distribution is plotted in a Preisach plane, using the coercive field *H_c_* = (*H* − *H_r_*)/2 and the interaction field *H_u_* = −(*H* + *H_r_*)/2 set of axes. However, since interacting magnetic nanowire arrays are systems that do not obey the two necessary conditions of the classical Preisach model (i.e., the congruency and wipe-out conditions [46,47]), they cannot be considered as sets of hysterons. This yields two important consequences for the analysis of their FORC distribution. First, the presence of a positive distribution at an (*H_c_, H_u_*) point is not necessarily related to the magnetization reversal of an entity with a coercivity value equal to *H_c_.* In the case of arrays of interacting single domain magnetic nanowires, it is due to the multiplicity of their magnetization reversal occurrences. Resulting typical examples are the extreme cases of the wishbone shape and the distribution extension along the *H_c_* axis, arising from a nanowire coercivity distribution respectively with a uniform [31,36] and non-uniform [37] interaction field, as well as the intermediate cases depending on these distribution relative intensity, width, and shape [48]. The second consequence is the possible presence of negative distribution values (ignoring those induced by experimental noise in the FORCs). Those negative regions emerge from the crossing of some FORCs and/or a lower susceptibility of the inferior FORC compared to the next superior one [37]. For nanowire arrays, since they are not directly related to a magnetization reversal occurring in this (*H_c_, H_u_*) region and typically of low amplitude, only the positive FORC distributions are commonly plotted in the FORC diagrams. However, for result completeness, we kept their representation in the experimental FORC diagrams of the modulated nanowire arrays investigated in this study.

We completed the experimental characterization with micromagnetic simulations. Using the free software MuMax^3^ (version 3.9.3, Ghent University, Belgium) [49], single Ni nanowires with a sharp diameter modulation were constructed (saturation magnetization *M_s_* = 480 kA/m, exchange coupling constant *A* = 9 pJ/m, cell size of 2.5 × 2.5 × 10 nm^3^ (except for *L* ≥ 5.6 μm, for which we used 20 nm as the axial cell extension). Both nanowire segments were either positively or negatively axially saturated, and the created demagnetization field around the nanowire was recorded after relaxing the magnetization without applied field.

## 3. Results

### 3.1. Nanowire Morphology

**Figure 1:**
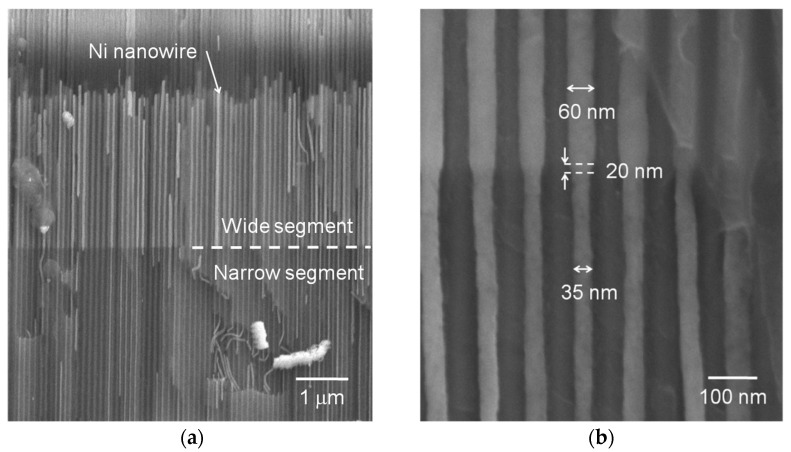
Representative SEM cross-section micrographs of the Ni diameter-modulated nanowire arrays. (**a**) Global view, showing the high filling factor and the constant height diameter modulation; (**b**) Zoom on the abrupt diameter modulation region. Note that for the filled pores, the magnetic material completely occupies the pore space until the nanowire top extremity, i.e., the Ni is in direct contact with the alumina walls.

We first verified the geometrically-modulated nanowire morphology through SEM (Figure 1). The nanowire array cross-section micrography shows a high filling factor and a small length distribution. Note that this nanowire length distribution is restricted to the wider segment, which is located on top of the narrow one (Figure 1a). Furthermore, the top extremity of the wide nanowire segments is the only one that is not perfectly aligned with their neighboring nanowires: both the bottom extremity of the narrow segment and the diameter modulation occur at the same height for all nanowires in the array. This geometrical constant modulation height denotes the capacity of the three-step anodization technique to obtain a high-quality diameter-modulated array. A zoom on the modulation region permits the observation of the abrupt yet slant diameter modification, extending over a (20 ± 10) nm-long region (Figure 1b).

### 3.2. Experimental Magnetic Behavior

#### 3.2.1. Major Hysteresis Curves

**Figure 2:**
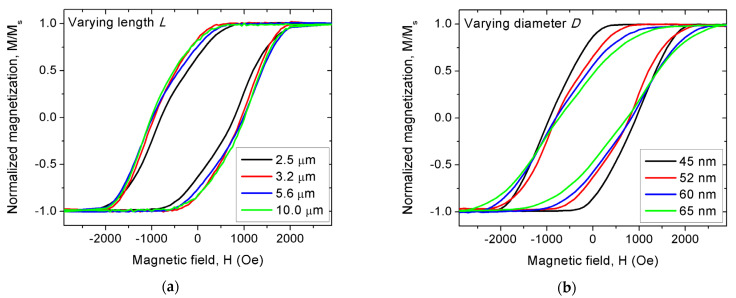
Major modulated Ni nanowire array hysteresis curves. (**a**) Varying the wide segment length *L* between 2.5 to 10.0 μm with fixed diameter *D* = 52 nm; (**b**) Varying the wide segment diameter *D* between 45 to 65 nm with fixed length *L* = 2.5 μm. Note that the arrays named *L* = 2.5 μm and *D* = 52 nm represent the same sample. The bottom nanowire segment was kept as (36.0 ± 0.3) nm wide and (3.4 ± 0.2) μm long.

All major hysteresis curves present a sheared shape, typical of nanowire arrays driven by shape anisotropy and large interaction field [16,23] (Figure 2). For the varying wide segment length, all nanowire arrays saturate near 2000 Oe, exhibiting coercivity and reduced remanence values ranging from 790 to 1020 Oe and 0.64 to 0.84, respectively. Except for both a lower coercivity and remanence in the smallest case (*L* = 2.5 μm), no clear trend is observed (Figure 2a). On the other hand, increasing the wide segment diameter induces an increasing saturation field (reaching up to 2500 Oe) and a reducing remanence, decreasing from 0.86 down to 0.46 (Figure 2b). In first approximation, the global increase of the demagnetizing interaction field, due to the increased magnetic volume, explains the observed tendency.

#### 3.2.2. First-Order Reversal Curve (FORC) Results

As previously explained, to more accurately probe the influence of the diameter modulation on the Ni nanowire array magnetization reversal, their FORC distribution has been calculated from a complete set of FORCs. In all cases, the measured FORCs are displayed in the inset of the array FORC result. Additionally, due to the presence of shallow negative values in the FORC distribution, they are represented in the FORC result as dark blue, while positive values range from light blue (null distribution) to red (maximum value). Here, the relative amplitude of the distribution values is less than 10% of the positive amplitude. Note that for all investigated modulated nanowire arrays, no reversible magnetic behavior occurs at the FORC curve beginning, since the susceptibility at *H_r_* is null. Therefore, the minor features visible along the *H_u_* axis are numerical artifacts that the FORC extrapolation did not completely achieve to suppress. Similarly, slight deviations around the FORC distributions are consequences of the experimental noise in the FORCs and should not be taken into account in the further analysis.

**Figure 3:**
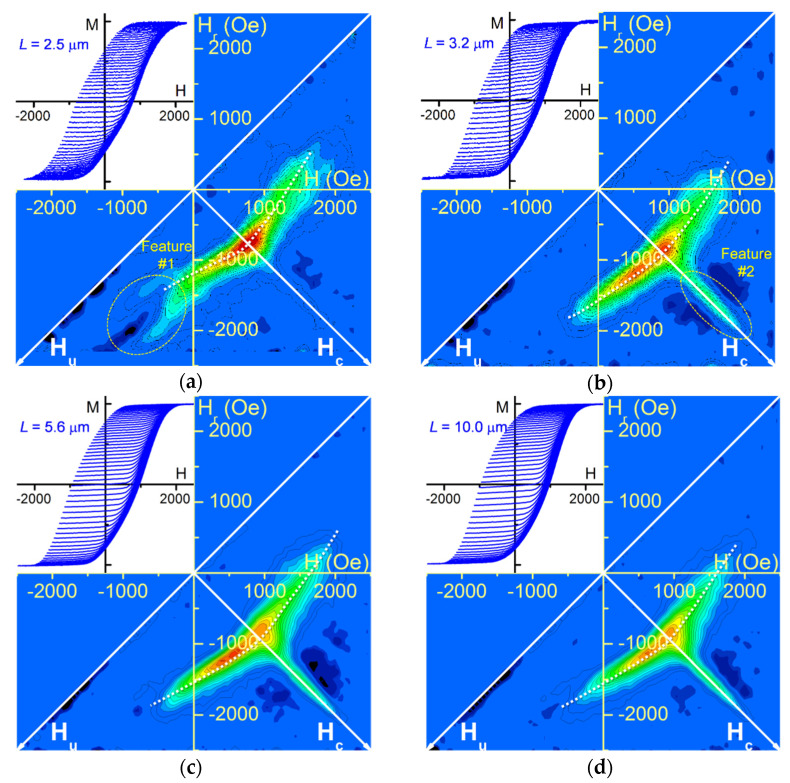
FORC results and FORCs curves (inset) for wide segment length *L* variation (*D* = 52 nm) (**a**) *L* = 2.5 μm; (**b**) *L* = 3.2 μm; (**c**) *L* = 5.6 μm; (**d**) *L* = 10.0 μm. Negative values are represented as dark blue, while positive values range from light blue to red. White dashed curves emphasize the FORC distribution curvature.

All FORC results for the wide segment length variation present a curved shape similar to the diameter-modulated Ni nanowire arrays previously investigated [39]. Rather than remaining parallel to the interaction field axis *H_u_*, the distribution elongation is closer at its extremities than at its middle point (located on the *H_c_* axis) (Figure 3). Nevertheless, for the shortest length (*L* = 2.5 μm), the FORC distribution exhibits an additional feature creating a fork structure at the bottom of the main FORC distribution, constituted of two positive branches (visible in cyan) separated by a thin negative region (denoted feature #1, see Figure 3a). This feature is uncommon for nanowire array FORC distribution but has been already observed for the shorter wide segment (*L* = 1.4 μm) of similar modulated Ni nanowire arrays [39]. Note that this fork shape was not present for the previously fabricated *L* = 3.2 μm array, in agreement with the currently obtained result.

Furthermore, they all exhibit a non-negligible extension on the coercivity field axis *H_c_* (denoted feature #2, see Figure 3b). As previously mentioned, this extension is characteristic of experimental FORC results of nanowire arrays with an axial easy magnetization axis, even cylindrical with a uniform diameter [32,34,35,36]. However, it should not be taken as a distribution of high coercivity nanowires, neither as the result of the magnetization reversal of individual nanowires with a *H_c_* switching field. In this case, the extension appearance is caused by the lower interaction field intensity felt by the nanowires located near the array border [37]. This characteristic appears to be clearly more prominent for lengths above 3.2 μm compared to the relative intensity of the main curved distribution (see Figure 3b–d), as well as better defined (less noisy). It is important to remember that both the relative amplitude and definition of the *H_c_* extension are directly related to the multiplicity of the magnetization reversal of the individual magnetic entities of the system. For example, using a higher field resolution (lower ΔH and ΔH_r_ field intervals) reduces this multiplicity, which diminishes the extension amplitude until it disappears in a noisy-like result [37]. Here, since the ΔH and ΔH_r_ field intervals were maintained constant for all FORC measurements while the hysteresis curves exhibit similar coercivity, the multiplicity of reversal occurrences should be modified by other factors. Finally, it is interesting to note that, through a careful examination of these FORC results, they all present a negative distribution just above the *H_c_* extension (i.e., in the negative *H_u_* quadrant). This is in addition to the one located below (i.e., in the positive *H_u_* quadrant, more or less pronounced depending on the samples), which is expected to appear together with the positive extension [37].

**Figure 4:**
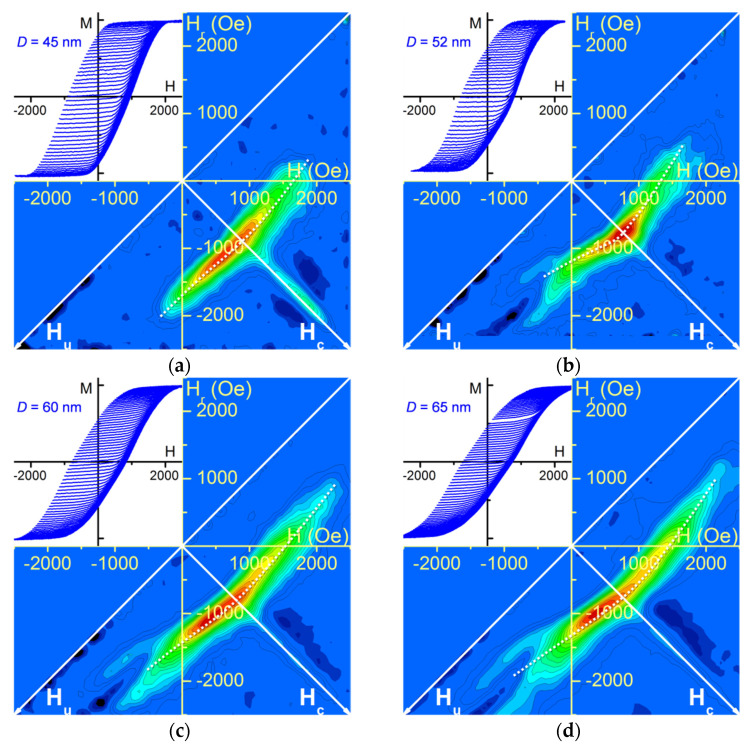
FORC results and FORCs curves (inset) for wide segment diameter *D* variation (*L* = 2.5 μm) (**a**) *D* = 45 nm; (**b**) *D* = 52 nm; (**c**) *D* = 60 nm; (**d**) *D* = 65 nm. Negative values are represented as dark blue, while positive values range from light blue to red. White dashed curves emphasize the FORC distribution curvature. The arrays named as *L* = 2.5 μm (Figure 3a) and *D* = 52 nm (Figure 4b) correspond to the same sample, but are shown in each series for consistency.

Similar to the FORC results obtained for different *L*, the different wide segment diameters *D* also yield a curved main FORC distribution, completed by a more or less distinguishable extension along the *H_c_* axis topped by a negative region (Figure 4). Furthermore, a clear fork shape at the main FORC distribution bottom appears for wide segment diameter *D* above 52 nm. While inexistent for the lower diameter (*D* = 45 nm), it becomes more pronounced while increasing the wide segment diameter. Analogously to the trend observed in Figure 3 results, the *H_c_* extension relative amplitude and sharpness is higher in the absence of a fork feature (Figure 4a).

### 3.3. Simulated Demagnetizing Magnetic Field

To better understand the different interaction fields that originated inside the modulated nanowire arrays, we simulated the stray field arising from a single nanowire (denoted as source nanowire). We focused on the axial and radial magnetic field where a first neighboring nanowire would stand, i.e., at a 105 nm distance from each nanowire center. 

**Figure 5:**
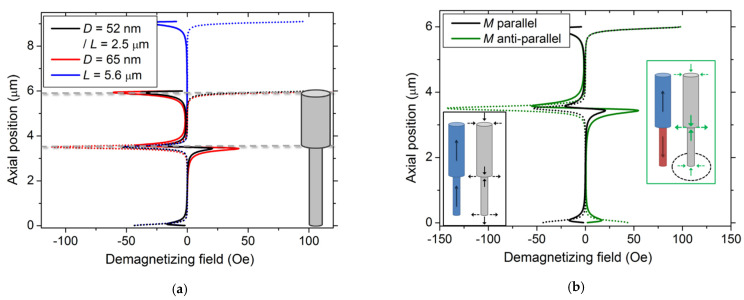
Simulated demagnetizing field of a single modulated source Ni nanowire, along a neighboring nanowire located at a 105 nm distance. Both the axial (solid line) and radial (dot line) components are shown. Positive demagnetizing field values refer to upward and inward directions, respectively. (**a**) Fully upward magnetization; (**b**) Comparison between source segments with parallel (both upward) and anti-parallel (narrow segment downward) magnetization (*D* = 65 nm). Insets: Schematic of the axial (solid arrows) and radial (dotted arrows) components of the stray field (black and green arrows) of the source nanowire (colored) around the neighboring nanowire (gray).

For a fully saturated nanowire, we can observe three main regions of interest, as expected (Figure 5a). First, the axial field is opposite to the magnetization of the source nanowire at both extremities, while at the diameter modulation, a parallel (positive)/anti-parallel (negative) field is induced (see solid arrows in the black inset, Figure 5b), with amplitude in-between those at the wide and narrow segment extremities. These axial components of the stray field increase with the segment diameter but remain constant with its length. A similar amplitude dependence upon the segment geometry is observed for the radial component. In this case, it points toward the source nanowire at the bottom and the modulation, while is directed inward and is considerably larger at the top extremity. In all regions, the radial component amplitude overcomes the axial one. 

However, inverting the magnetization direction of one of the segments modifies both the direction and the amplitude of the stray field. When reversing the narrow segment, only the field at its free extremity inverses (both components) (encircled in green inset, Figure 5b). However, we observed that the field amplitude at the modulation increases by about a factor around 2.7 (for both axial and radial components), thus overcoming the amplitude at the wide segment free extremity. 

Finally, we can resume our observations stating that: (1) the stray field amplitude only depends on the segment diameter and not on its length; (2) the radial component is larger than the axial one at the modulation; (3) both components increase when each segment magnetizations are opposite; and (4) the narrow segment magnetization direction only governs the stray field at its free extremity, while the wide one imposes the stray field directions at the modulation.

## 4. Discussion

All experimental FORC results obtained for diameter-modulated nanowire arrays exhibit a curved FORC distribution, as already previously observed [39], along with an unexpected negative region located above the *H_c_* axis. However, they differ between each other based on two distinctive features: a fork structure located at the bottom of the main distribution (feature #1) and the clearness of the *H_c_* extension (feature #2). One can note that in the array set investigated in this study, the presence of the fork shape is combined with a low *H_c_* ridge, whereas none of the arrays exhibiting a prominent one presents a fork feature. Therefore, when analyzed as a whole, it suggests the separation in two possible distinct processes for the magnetization reversal of nanowire arrays with a single diameter modulation. 

The transition from one process to the other seems to be related to the wide segment length vs. diameter aspect ratio *L*/*D* (the narrow segment dimensions being kept constant). The lack of fork structure combined with a distinct extension occurs for *D* = 45 nm and *L* ≥ 3.2 μm nanowire arrays, for which the aspect ratio ranges from 55 up to around 200. On the opposite, arrays with a lower aspect ratio (below 48, i.e., *D* ≥ 52 nm with *L* = 2.5 μm) may present a modified magnetization reversal, which yields a fork pattern while decreasing the *H_c_* ridge. Therefore, in addition to the asymmetric magnetic interaction field introduced in the nanowire arrays by the diameter difference among the nanowire extremities, the nanowire wide segment shape anisotropy could govern further complex magnetic behavior as the magnetic field is swept.

### 4.1. Higher Wide Segment Aspect Ratio (L/D above 55)

**Figure 6:**
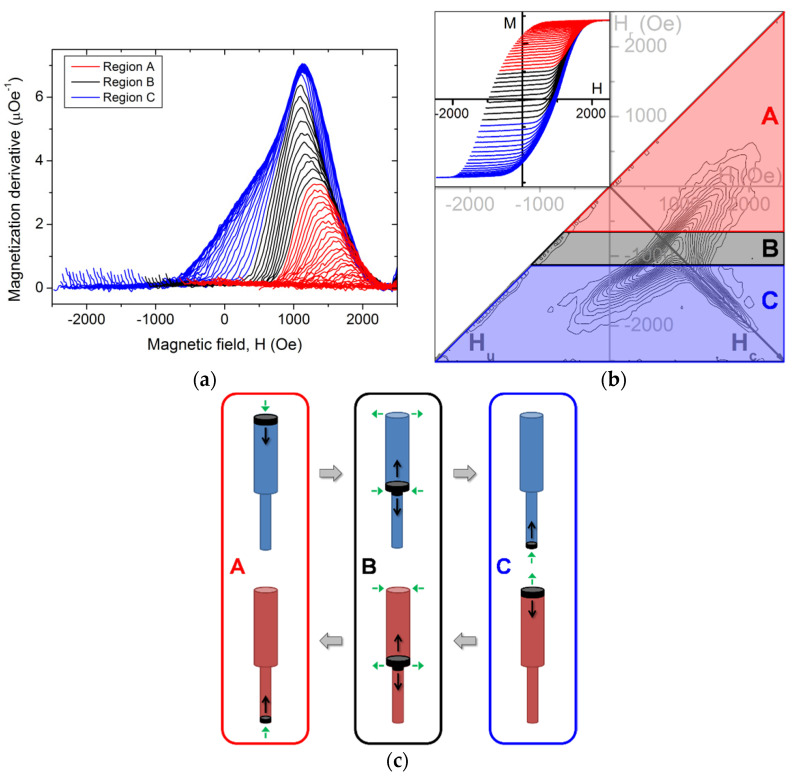
FORC data from nanowire array with *L* = 10.0 μm, divided according to the magnetization reversal processes occurring while reducing the magnetic field. (**a**) Derivative of the FORCs; (**b**) Respective FORC diagram, with the FORCs in inset; (**c**) Schematic representation of the proposed magnetization reversal processes from upward to downward (blue nanowires, top) and downward to upward (red nanowires, bottom) occurring while the applied field is decreasing until *H_r_* and increasing back to the saturation, respectively. The green arrows represent the involved interaction field acting on the nanowire, while the black regions and arrows respectively indicate the DW nucleation region and propagation direction.

Modulated nanowires with a length/diameter high aspect ratio of the wide segment have a high magnetic anisotropy, favoring an axial magnetization alignment and thus a magnetic single domain state for the nanowires. From the magnetization susceptibility ∂M/∂H, the FORC distribution can be divided into three distinct regions, based on their different magnetic behaviors (Figure 6).

The first region, denoted A (in red), is characterized by a linearly increasing susceptibility of the major hysteresis curve, indicating that the magnetization reversal is self-promoting (Figure 6a). It ranges from the positive saturation until a reduced magnetization of 0.35, i.e., until around a third of the magnetic volume is reversed (see inset, Figure 6b). The related FORC distribution presents an elongated shape but not parallel to the *H_u_* axis (Figure 6b). According to previous investigations, the first nanowires are thought to inverse through the nucleation of a DW at the nanowire extremity where the demagnetizing interaction field is the highest, i.e., at the wide segment one (see Figure 5 and Figure 6c, A process, top). The magnetic DW propagation would occur until reaching a negative single domain state since no reversible magnetic behavior is observed at the reversal fields (*H_r_ = H_u_*). The switching back process is similar, except that the DW nucleation would now happen at the narrow segment extremity, due to the lower interaction field at this point (Figure 6c, A process, bottom). Therefore, we assume that the axial component of the interaction field at both nanowire extremities governs the magnetization reversal process until a third of the nanowires are reversed.

Decreasing further the applied field yields an abrupt increase of the susceptibility (Figure 6a). This behavior continues until it remains a third of the array magnetization to reverse before the negative saturation (region B, in black; see inset in Figure 6b). One hypothesis is that since the nanowire array magnetization is low, the influence of the axial component of the long-range dipolar field, which is favoring a DW nucleation at the nanowire extremities, is overcome by another phenomenon. One possibility arises from the radial component of the stray field at the diameter modulation, as observed in Figure 5. For a saturated nanowire array, these radial stray fields cancel one with each other over the whole array. However, when considering two neighboring nanowires with opposed magnetization, their radial stray fields sum in the space between them. Therefore, the transition between regions A and B may arise from a change in the magnetization reversal, passing from a nucleation at the nanowire wide extremity to being induced by a lateral (radial) field. Despite the lower field amplitude, we favor a magnetization reversal initiating at the modulation instead of at the wide segment extremity since the system is more sensitive to a magnetization perturbation in this zone (Figure 6c, B process, top). This process seems to happen when the magnetization of most neighboring nanowires is opposite, suggesting a short-range influence. Note that this phenomenon occurrence depends on several factors, such as the modulation sharpness and its constant height along the nanowires. While the related FORCs and their derivatives seem similar to those yielding region A, the FORC distribution in region B is more complex to analyze. Due to the lack of a clear modification in the FORCs derivatives, it is probable that the switching back of the magnetization also occurs through radially-favored magnetization reversal mechanism (Figure 6c, B process, bottom), giving rise to the large FORC distribution in this region (Figure 6b). After reaching a certain magnetization value, further magnetization reversals return to be driven by an axial field at the narrow segment extremity (Figure 6c, A process, bottom).

Finally, the third FORC region (C, in blue) seems to be similar to region A, with a continuous decrease of the *H_c_* coordinate as increasing the |*H_u_*| one (Figure 6b). The magnetic susceptibility is reducing while decreasing the applied field, through magnetization inversion via DW nucleation at the most favorable point, i.e., now at the narrow segment extremity (Figure 6c, C process, top). The main FORC distribution denotes the beginning of the reversal back to an upward magnetization, thus through a DW nucleation at the wide segment extremity (Figure 6c, C process, bottom). The derivatives of the region C FORCs collapse at the same field as the regions A and B transition (≈700 Oe, see Figure 6a), i.e., when the nanowire array passes from an axially driven magnetization reversal to a radial one. Therefore, it suggests that the nanowire switching is followed by the radial stray field process, until returning again to a nanowire extremity DW nucleation, now the narrow one (Figure 6c, processes B and A, bottom). 

Due to the large width of the FORC distribution, accurate values of the axial interaction field were not extracted, which could be done following the procedure described in [39]. However, qualitative observation of the FORC result curvatures for the nanowire arrays with an aspect ratio above *L*/*D* = 55 (see Figure 3b–d and Figure 4a) yields that the interaction field difference between the array top and bottom is effectively lower for the smallest diameter (*D* = 45 nm), in agreement with the simulated stray field analysis. However, this difference appears to increase, as well as the field amplitude, when increasing the wide segment length *L*. Even if these values are not affected by the length in a single nanowire, the overall increase of magnetic volume in the nanowire array may account for this behavior. 

As previously mentioned, the main FORC distribution of the arrays where the wide nanowire segments have a high shape anisotropy is completed by a strong and sharp extension along the *H_c_* axis (feature #2, see Figure 3b). This common feature arises when both the individual switching field and the felted demagnetizing interacting field differ among the magnetic entities. Physically, several causes can produce an analogous feature, such as the lower mean interaction field intensity at the array lateral border [37] or a predominant local magnetostatic interaction field [48]. Here, we may also consider the radial stray field as a source of interaction field non-uniformity. Since it has been shown that a higher switching occurrences multiplicity yields a well-defined ridge, it may indicate that the number of nanowire magnetization reversals occurring in a ΔH per ΔH_r_ area greatly varies among subsequent FORCs. In other words, it suggests that a small difference in the initial magnetic structure (at *H_u_* = *H_r_*) will greatly affect the magnetization evolution while returning to the positive saturation.

Another related observation can be done concerning the unexpected negative region lying above the *H_c_* extension. From the FORCs derivative analysis, it arises around *H* ≈ 1500–2000 Oe from the crossover of some inferior FORCs (with a higher susceptibility) above the superior ones, which have a lower susceptibility. We hypothesize that since the approach to the (positive) saturation susceptibility depends on the reached region during the field decreasing, there is a superposition of the three DW nucleation possibilities proposed in Figure 6c, instead of a sharp regime switch.

### 4.2. Lower Wide Segment Aspect Ratio (L/D below 48)

**Figure 7:**
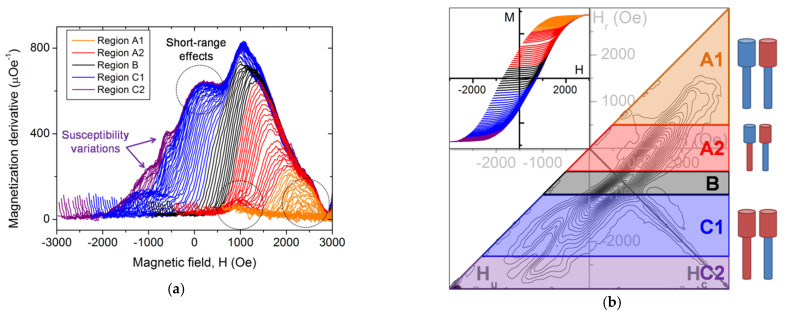

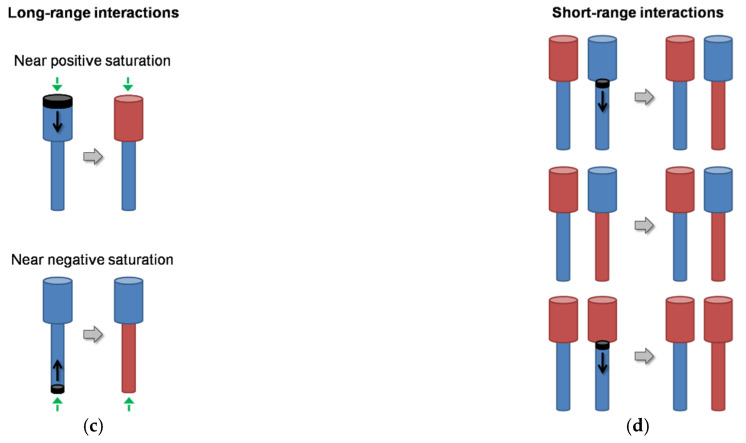
FORC data from nanowire array with *D* = 65 nm, divided according to the magnetization reversal processes occurring while reducing the magnetic field. (**a**) Derivative of the FORCs. Both the susceptibility variations and the short-range effects (encircled) are highlighted.; (**b**) Respective FORC diagram, with the FORCs in inset and a possible magnetic configuration depicted on the left; (**c**,**d**) Schematic representations of the possible magnetization reversal processes occurring while the applied field is decreasing from upward magnetization (blue segments) to downward (red segments), arising from long-range and short-range interactions, respectively. The green arrows represent the involved interaction field acting on the nanowire, while the black regions and arrows respectively indicate the DW nucleation region and propagation direction.

When decreasing the length/diameter aspect ratio, the shape anisotropy reduces simultaneously. This may give rise to a multidomain state, where each segment is axially magnetized but in opposite directions. Comparing the magnetization susceptibility behavior for the lower aspect ratio (below 48) with the result for the higher ones, we can recognize some similarities, such as its asymmetry and a narrow maximum prior to the sharp decrease (Figure 7a). However, the ∂M/∂H behavior that is identified as region C for *L*/*D* above 55 is much more complex. It exhibits at least two clear plateaus, instead of a smooth increase. Furthermore, a small kink is perceptible in what is attributed to region A when *L*/*D* > 55, just before reaching the magnetization saturation. Therefore, based on these features, we divide both regions A and C discussed in Section 4.1 into two sub-regions: A1, A2, and C1, C2, respectively (Figure 7a,b).

The exact and complete description of the nanowire array magnetization reversal in this case is beyond the scope of this work. Moreover, the richness of the different magnetic behaviors shown by the susceptibility and the FORC result, especially when compared to uniform diameter Ni nanowire arrays, tends to indicate that small experimental variations in the array can lead to some important modifications of their magnetic behavior. Therefore, here, we will limit ourselves to propose possible magnetization reversal mechanisms. Note that we assume that the magnetic behavior in the middle region (B, in black), where the global magnetization is low, continues to be driven by short-range consequences of the radial interaction field. 

We observe that it is possible to divide the susceptibility behaviors into two groups. On one side, those that are shifting depending on the reversal field values, which lead to a feature on the FORC distribution. Therefore, they are assumed to arise from the axial component of the interaction field, but as long-range consequences, i.e., from the average nanowire array magnetization. This is the case of the main curved feature, which is similar to the ones already discussed in Section 4.1, as well as the additional fork branch located at its bottom. On the other side, the susceptibility curves reveal several magnetic features that do not appear on the FORC distribution because they always occur at the same field value. In this case, it suggests a short-range effect, where the influence of the near neighbors is preponderant on the global nanowire array interaction field. The most important are located around 0 Oe, 1000 Oe, and 2400 Oe (circled in Figure 7a).

Opposite to what happens for high *L*/*D* aspect ratio nanowires, the long-range interaction may inverse only one segment: the wider one when leaving the positive saturation but the thinner one while reaching the opposite saturation, similarly to the A and C behaviors, respectively (Figure 7c). Consequently, not only that the average axial interaction field favors two-domain nanowires, but these magnetic structures will promote an anti-parallel magnetization alignment of neighboring multidomain nanowires (some examples are depicted in Figure 7d, which may not all occur during the magnetization reversal). Based on these observations, we propose that when the applied field reduces from the positive saturation, the demagnetizing field reverses the wide segment of some nanowires (Figure 7c, top), which will induce the reversal of the narrow one of a neighboring nanowire when reducing further the external field (Figure 7d, top). This process tends to form small clusters of anti-parallel multidomain nanowires. These highly stable magnetic structures may explain the modification of the FORC distribution inclination (with respect to the *H_u_* axis) dividing the A1 (in orange) from the A2 region (in red) if the magnetic susceptibility bump at 1000 Oe arises from their formation (see Figure 7b). In A1, all multidomain nanowires are down/up at *H_r_* (as in Figure 7c, top), thus saturating back with a lower field than in A2, where anti-parallel multidomain nanowire clusters require a higher field to saturate positively (as in Figure 7d, middle).

Similarly, it is plausible that just before reaching the negative saturation, the nanowire array contains both multidomain nanowires (up/down and down/up), together with saturated ones. When inverting the field sweeping direction from this situation, two kinds of nanowires for which the interaction field will reverse the wider segment magnetization are now present: (1) the single domain nanowires (with magnetization pointing down), but also (2) the multidomain ones with the narrower segment already pointing along the field sweeping direction, i.e., positive (Figure 7d, bottom). Since the multidomain nanowires should switch at a lower field than the single domain ones, their presence in the array at the reversal field yields the first branch of the fork feature. Based on this hypothesis, the FORC measurement indicates that the nanowire array contains these multidomain nanowires in the field interval of −1300 to −2500 Oe (C1 region, in blue). The presence of negative FORC distribution values between the fork branches is simply a consequence of the FORC curve crossover resulting from the different single/multidomain nanowire proportions at the curve beginning in this field interval. Hence, the second branch can be considered as a unique feature composed of a negative/positive distribution. 

Additionally, as already pointed out in Figure 5b, the multidomain configurations create a stronger demagnetizing field around the modulation of the neighboring nanowires. The axial component of this short-range interaction is thought to reverse one segment of this neighboring nanowire. Since several combinations are possible, a more exhaustive study of this aspect is required to identify those occurring around 0 Oe, 1000 Oe, and 2400 Oe, but they may explain the minor susceptibility variations in the C2 region (in purple, see Figure 7a).

Finally, concerning the positive FORC extension along the coercivity axis and the unexpected negative region above it, we can extend the discussion presented in Section 4.1 to the nanowire arrays with a lower *L*/*D* ratio. In this case, the extension greatly reduces in amplitude, suggesting that the amount of magnetization reversal between subsequent FORCs in a given ΔH interval varies less than for *L*/*D* > 55. One hypothesis is that the presence of multidomain nanowires yields a greater “stiffness” to the system, i.e., a magnetization reversal less dependent on small differences in the original magnetic structure, than when all the nanowires remain single domain. However, similarly to the high aspect ratio case, the nearby negative region would arise from a superposition of the different DW nucleation possibilities during the magnetization evolution of experimental nanowire arrays.

## 5. Conclusions

In summary, the magnetization reversal of geometrically modulated magnetic nanowires is governed both through long-range and short-range interaction field consequences. The axial long-range component yields an asymmetric magnetization reversal mechanism, since the extremity at which the DW nucleates depends on the interaction field direction (and thus the magnetization signal). This seems the only magnetization reversal mechanism occurring for nanowire arrays characterized by a high wide segment aspect ratio. In this case, the nanowires are thought to remain single domain during the whole magnetization reversal. On the opposite, a low wide segment aspect ratio creates a multidomain magnetic structure for the nanowires, which consequences on the nanowire array magnetization reversal are evidenced by a characteristic fork feature on the FORC diagrams along with a weaker positive extension along the coercivity axis. On the other side, both components (radial and axial) of the short-range interaction field act on the neighboring nanowires, but always near the segment modulation. For all nanowire shapes, the radial component interferes in the reversal around null magnetization, while the axial one promotes several different magnetic configurations for low wide segment aspect ratio arrays. In both cases, these magnetic behaviors are not directly perceptible in the FORC diagrams, but in the FORC curve derivatives.

## Data Availability

The data presented in this study are available on request from the corresponding author.
